# Direct detection pays off for electron cryo-microscopy

**DOI:** 10.7554/eLife.00573

**Published:** 2013-02-19

**Authors:** Nikolaus Grigorieff

**Affiliations:** 1 **Nikolaus Grigorieff** is at the Department of Biochemistry and the Howard Hughes Medical Institute, Brandeis University, Waltham, Untied Statesniko@brandeis.edu

**Keywords:** Electron microscopy, Image processing, T. thermophilus, Direct electron detectors, ribosome, Bayesian, S. cerevisiae

## Abstract

Improved electron detectors and image-processing techniques will allow the structures of macromolecules to be determined from tens of thousands of single-particle cryo-EM images, rather than the hundreds of thousands needed previously.

**Related research article** Bai X, Fernandez IS, McMullan G, Scheres SHW. 2013. Ribosome structures to near-atomic resolution from thirty thousand cryo-EM particles. *eLife* **2**:e00461. doi: 10.7554/eLife.00461

**Image** Cryo-EM image of 70S ribosomes with vectors indicating movement during beam exposure

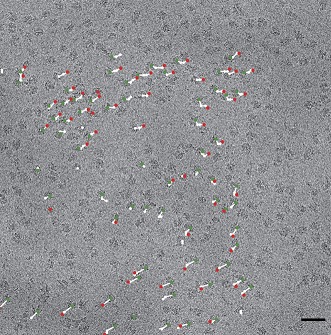

Electron cryo-microscopy (cryo-EM) is a technique that can be used to study the three-dimensional structure of macromolecules and their assemblies under close-to-native conditions. First used to study hydrated protein crystals in 1974 (Taylor and Glaeser, 1974), it was later extended to non-crystalline (single particle) preparations of viruses (Adrian et al., 1984). In cryo-EM, a preparation of the macromolecule to be studied is frozen in a thin layer of ice, and this layer is imaged in an electron microscope. The single-particle approach to cryo-EM, which emerged in the late 1970s (Frank et al., 1978; van Heel and Frank, 1981), offered the possibility of being able to determine the atomic structures of cellular machinery without the need for crystals, as required for X-ray crystallography.

To obtain a macromolecular structure using single-particle cryo-EM, many molecules need to be imaged, each displaying a particular view. In a process similar to tomography, the different views can be computationally assembled into a three-dimensional image—the structure of the molecule. However, unlike tomography, the angles describing the views are not known *a priori* and have to be determined by aligning the images to a reference structure. Any errors in the alignment will reduce the resolution of the final structure.

A deciding factor in single-particle cryo-EM is the signal-to-noise ratio in the images, which determines how many images have to be included in the data set to achieve high resolution. The signal-to-noise ratio also influences the alignment error. (The number of images required and the alignment error are not completely independent, and it is possible to make up for alignment errors by collecting more images). Particles with a high molecular mass, such as icosahedral viruses, generate strong contrast (signal) in the images. Moreover, since these viruses are highly symmetric, the number of images needed to determine their structure is smaller than that needed for less symmetric structures. Therefore, until recently, single-particle cryo-EM had fulfilled its promise to yield atomic models without crystals only in the special case of large, symmetrical assemblies such as icosahedral viruses.

Now, in eLife, Xiaochen Bai, Israel Fernandez, Greg McMullan and Sjors Scheres of the MRC Laboratory of Molecular Biology (LMB) in Cambridge report that single-particle cryo-EM can be used to determine the structure of smaller, asymmetrical assemblies with near-atomic resolution (Bai et al., 2013). They demonstrate this by determining the structure of the 80S ribosome with a resolution of close to 4 Angstroms.

Single-particle cryo-EM is ideally suited to the large macromolecular assemblies that drive many of the complex processes that take place inside cells. It is often difficult to make crystals of these assemblies because they tend to occur in different conformations that are difficult to discriminate biochemically, and because they are often too fragile to survive the process of crystallization. The possibility of using single-particle cryo-EM to determine atomic structures was described over a decade ago by Henderson and Glaeser, who also estimated the maximum contrast that could be achieved in cryo-EM images before the sample is destroyed by the electron beam (Henderson, 1995; Glaeser, 1999).

According to these calculations it should be possible, under ideal conditions, to determine the atomic structure of the sample from just a few thousand low-dose images. In reality, however, far greater numbers have been required—between several hundred thousand and a few million imaged molecules - to allow the structure of larger symmetrical complexes to be determined (see, for example, Mills et al., 2013). Moreover, it has not been possible to achieve comparable resolution in experiments on smaller, asymmetrical particles. For example, the previous record value for the resolution achieved in cryo-EM studies of 80S ribosomes was about 6 Angstroms, which is insufficient for determining the atomic structure, despite the fact that about 1.4 million images were needed to achieve it (Armache et al., 2010).

Exploiting the full potential of single-particle cryo-EM involves reducing the loss of contrast and the addition of noise during the imaging process (Figure 1). The two main sources of contrast loss, and hence lowered resolution, are the electron beam causing the particles to move during the experiment, and the low efficiency of the detectors used to record the images. Both problems can be addressed with a new generation of devices that detect the electrons directly. Previously researchers have used indirect devices such as scintillators coupled with CCD cameras (in which the electrons are converted into photons of light before being detected). Using the fast read-out rate of the new direct CMOS detectors, Bai et al. recorded movies of the ribosomes, which allowed them to follow the movement caused by the electron beam, and then correct for it computationally. The resulting ribosome images were of such high quality that they could be aligned with sufficient accuracy to obtain near-atomic resolution from only about 30,000 images. The alignment was performed with a new algorithm based on Bayesian statistics (Sigworth, 1998) and further developed by the authors. The LMB work also highlights another advantage of the single-particle approach: it is possible to identify different conformational states in a sample containing a mixture of states. In other words, Bai et al. were able to ‘purify' their sample in silico.

**Figure 1.**
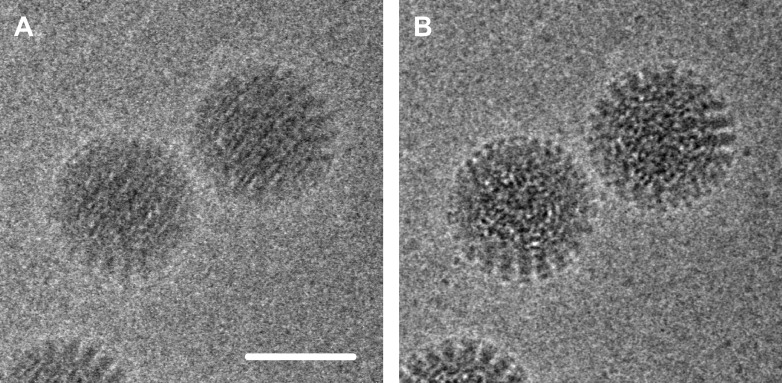
Electron micrograph of double-layered rotavirus particles frozen in a thin layer of amorphous ice. The image was recorded by the author and co-workers using the DE-12 direct electron detector (Direct Electron, San Diego, United States) in movie mode at 40 frames per second. In panel A, 60 frames have been averaged without alignment, resulting in an image that is blurred due to beam-inducted movement. In panel B the frames have been aligned to compensate for this movement, which results in an image with significantly reduced blurring and improved contrast. The alignment method used here involved tracking the movement of the particles (Brilot et al., 2012); the alignment method used by Bai et al. used additional statistics to predict the movement of the particles caused by the electron beam. Scale bar = 50 nm.

FIGURE CREDIT: AXEL F BRILOT AND NIKOLAUS GRIGORIEFF

The single-particle technique still has a way to go before it can become mainstream. Common standards in the field need to be established, such as common data formats and quality criteria (Henderson et al., 2012). There is also a need for national cryo-EM facilities to be maintained and made easily accessible for groups and laboratories that do not maintain their own equipment. More than a decade ago, Henderson and Glaeser optimistically predicted a bright future for cryo-EM, and now Bai, Fernandez, McMullan and Scheres have brought us a significant step closer towards realizing this future.
